# Synthesis of Zinc Oxide Nanorods from Zinc Borate Precursor and Characterization of Supercapacitor Properties

**DOI:** 10.3390/nano13172423

**Published:** 2023-08-25

**Authors:** Chikh Lefdhil, Safa Polat, Hüseyin Zengin

**Affiliations:** 1 Material Research and Development Centre, Karabuk University, 78050 Karabük, Turkey; 2 Nano Energy Laboratory, Karabuk University, 78050 Karabük, Turkey; 3 Metallurgy and Materials Engineering, Karabuk University, 78050 Karabük, Turkey; 4 Institute of Chemical Technology of Inorganic Materials (TIM), Johannes Kepler University, 4040 Linz, Austria

**Keywords:** zinc borate, leaching, ZnO, electrode, supercapacitor

## Abstract

The synthesis of zinc oxide (ZnO) was accomplished from zinc borate (Zn_3_B_2_O_6_) minerals to be used as electrodes in supercapacitor applications. The concentrations of obtained zinc (Zn) metal after treatment with hydrochloric acid (HCl) were determined by atomic absorption spectroscopy (AAS). Direct synthesis of ZnO on a nickel (Ni) foam surface was conducted by employing the hydrothermal technique using a solution with the highest Zn content. The results showed the successful synthesis of ZnO nanorods on the surface of Ni foam with an average wall size of approximately 358 nm. Cyclic voltammetry (CV) and galvanostatic charge–discharge (GCD) measurements revealed that the synthesized electrode exhibited battery-type charge storage characteristics, reaching a maximum specific capacitance of approximately 867 mF·cm^−^² at a current density of 2 mA·cm^−^². Additionally, the energy and power densities of the electrode at a current density of 2 mA·cm^−^² were calculated as 19.3 mWh·cm^−^² and 200 mW·cm^−^², respectively. These results exhibited promising performance of the single-component electrode, outperforming the existing counterparts reported in the literature.

## 1. Introduction

Supercapacitors are energy storage devices in which large amounts of electrical energy can be stored [1]. They have several advantages, such as fast charging and discharging times, long lifespan, and wide operating temperature range, making them suitable for various applications, including electric vehicles, portable electronic devices, and power tools [2,3,4]. Supercapacitors are divided into two categories, namely electrochemical double-layer capacitors (EDLCs) and pseudocapacitors (PCs). This classification is based on their charge storage mechanisms [5,6]. EDLCs store energy through the reversible attachment and detachment of ions at the electrode–electrolyte interface, while PCs rely on fast and reversible chemical reactions between electrolyte ions and electroactive materials [7]. Carbon materials with a two-dimensional high surface area are commonly used as the EDLC component, while metal oxides capable of redox reactions serve as the PC component [8,9,10]. Generally, EDLCs have a longer service life but lower specific energy storage capacity compared to pseudocapacitors [11]. Therefore, research has focused on developing composite materials that combine both characteristics and pseudocapacitive materials with a large surface area.

Among metal oxides, ZnO is a commonly used electrode material in supercapacitors. Supercapacitors function as electrodes using ZnO, undergoing oxidation and reduction reactions with ions present in the solution while also facilitating the adsorption of these ions on their surface to enable charge–discharge processes [12,13]. The most influential parameters affecting this process are its geometry and conductivity [14,15]. As a supercapacitor material, it possesses reasonably good conductivity (up to 230 S·cm^−1^) [16]. Its geometry is desired to have thin walls, facilitating ion diffusion. This aspect is diversified to further enhance performance. Consequently, ZnO metal oxide performs with a high specific capacity of approximately 978 mAh·g^−1^, exceptionally well as a supercapacitor electrode material [17,18,19]. In this context, Zhang et al. conducted the synthesis of ZnO with nano-rod geometry on a silicon wafer and found a specific capacitance (Cs) of 43 mF·cm^−2^ at a current density of 1 mA·cm^−2^ [20]. However, when they introduced ZnS as a dopant in the same study, they observed an increase in Cs value to 217 mF·cm^−2^. Kalpana et al. reported the fabrication of a ZnO/Carbon aerogel composite electrode, achieving a Cs value of 500 F·g^−1^ in a 6 M KOH solution [21]. Similarly, Jayalakshmi et al. prepared a composite electrode by incorporating carbon into ZnO. However, they could only achieve a low specific capacitance of 21.7 F·g^−1^ [22]. Selvakumar et al. observed a specific capacitance of 160 F·g^−1^ for ZnO/AC nanocomposite electrodes [23]. These results are considerably low compared to current electrodes. The main possible reasons for this are the use of relatively thick particle sizes for obtaining the active material and the utilization of a binder. It is suggested that having a thinner thickness for the active materials mounted on the current collector surface enhances electrical conductivity and facilitates ion diffusion.

Xiao et al. must have taken advantage of these predictions as they planned to create a core-shell structure by coating the carbon particle surface with ZnO in the form of a thin film [24]. They determined the specific capacitance (Cs) of the electrode to be 630 F·g^−1^ at a density of 2 A·g^−1^. Zhang et al., on the other hand, fabricated electrodes with thin-walled ZnO nanoplate geometry using the electrodeposition method without using a binder [25]. They later investigated the electrochemical performance of these electrodes by introducing graphene as an additive. While the pure ZnO electrode exhibited a specific capacitance of 118 F·g^−1^ at a current density of 3 A·g^−1^, the graphene-modified electrode showed a specific capacitance of 291 F·g^−1^. Jayachandiran et al. synthesized graphene oxide-modified ZnO nanoparticles using a specialized ultrasonication-assisted method [26]. It was observed that the particle size was approximately 80 nm, and the electrochemical performance of the synthesized nanoparticles was 312 F·g^−1^. These studies demonstrate that the specific capacitance (Cs) of pure ZnO remains around 200 F·g^−1^. Despite attempts to enhance it by avoiding the use of a binder and synthesizing the material at the nanoscale, the results were not sufficient, prompting the introduction of secondary additives. A similar observation was made for CuFe_2_O_4_, where it was found that CuFe_2_O_4_ exhibited a notably high Cs value without the need for additional additives when they were manufactured with a very thin thickness. Therefore, it is believed that the synthesis of ZnO in a pure, thin-walled geometry would offer significant advantages. Additionally, obtaining ZnO from a mineral-based source can offer a significant advantage in terms of sustainability. In conclusion, various metal oxide electrodes, including ZnO, have been prepared for supercapacitors up to this point. However, in this study, the direct utilization of a mineral as a zinc source and its synthesis on the binder-free current collector surface through a hydrothermal method will contribute uniqueness to the literature.

Building upon prior research, this study aims to directly synthesize ZnO on the surface of a current collector electrode at the nanoscale by extracting Zn from a zinc borate mineral source. The optimization of Zn concentration through extraction was performed, followed by the evaluation of the electrochemical performance of the resulting electrode. The syntheses were carried out on a Ni foam surface using the hydrothermal technique, employing a solution containing the highest Zn concentration. The synthesized products were analysed using various techniques such as Fourier Transform Infrared Spectroscopy (FTIR), X-ray diffraction (XRD), X-ray Photoelectron Spectroscopy (XPS), Brunauer–Emmett–Teller (BET), Scanning Electron Microscopy (SEM), and Transmission Electron Microscopy (TEM) to examine their chemical bonds, crystal structure, and microstructure. Electrochemical characterization involved Cyclic Voltammetry (CV) and Galvanostatic Charge–Discharge (GCD) measurements in a three-electrode system. Specific capacitance, energy density, and power density of the ZnO electrodes were calculated based on the electrochemical results. Finally, the obtained findings were compared with existing literature to provide further insights and evaluation.

## 2. Experimental

### 2.1. Materials and Method

The zinc borate mineral (2ZnO·3B_2_O_3_·3.5H_2_O) with a purity of 99% was purchased from Refsan A.S. Hydrochloric acid (HCl) with a concentration of 37% and deionized water with a resistance of 18.25 Mohm were used in the extraction process. Filtration was performed using 2.5 µm Whatman filter paper and glass centrifuge tubes. A reactor equipped with a stainless-steel outer wall and a Teflon lining was used for the hydrothermal synthesis. A current collector electrode consisting of Ni foam with a thickness of 1.6 mm and a density of 346 g·m^−2^, with a purity of 99.99%, was utilized. All products were used without further purification.

### 2.2. Leaching of Zinc Borate

The extraction of Zn metal from zinc borate was carried out through a simple leaching process. The main purpose of this process is the extraction of zinc from the ore. The amount of zinc extracted is dependent on the acidity level of the solution used for extraction. For this reason, the solution with the highest zinc concentration is determined first, and then the synthesis of zinc oxide from this solution is intended. Initially, a beaker containing 50 mL of deionized water was placed on a heater and gradually heated until the temperature reached 80 °C. At this temperature, 3 g of zinc borate was added to the mixture, followed by magnetic stirring at a speed of 360 rpm for 1 h. Afterward, 1 mL of HCl was gently added, and the mixture was stirred until it became clear (approximately 30 min). During this process, the reaction described in Equation (1) was expected to occur [27]:

2ZnO·3B_2_O_3_·3.5H_2_O + 4HCl + 6.5H_2_O → 2ZnCl_2_ + 6H_3_BO_3_ + 3H_2_O (1)

After this process, the beaker was transferred to an ice bath and left for 2 h to allow boric acid (H_3_BO_3_) to precipitate and separate from the solution. Subsequently, the white precipitates in the solution were first filtered through filter paper and then centrifuged at 3600 rpm for 15 min. The same process was repeated using 3 mL, 5 mL, 7 mL, and 9 mL of HCl. Then, the Zn concentration in the solution was measured using atomic absorption spectroscopy (AAS).

### 2.3. Production of Electrode by Hydrothermal Technique

**Figure 1:**
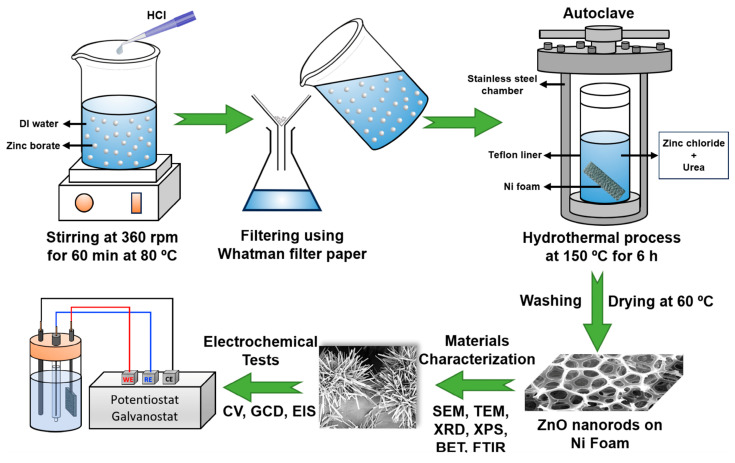
Schematic illustration of ZnO synthesis and characterizations.

The solution with the highest concentration of Zn after extraction was used for ZnO doping on the Ni foam surface. The production was carried out using the hydrothermal technique. The primary objective of this method was to create compounds by transforming dissolved Zn metal and precipitating ions (urea) in pure water under high pressure and low-temperature conditions, leading to the crystalline deposition of ZnO on a substrate surface [28,29]. The waiting time, temperature, and solution concentrations that influence crystal formation were determined based on our previous work and information from the literature [30,31]. The mechanistic formation of ZnO crystals likely occurred following Equations (1)–(4), as described below [32,33]. Firstly, 10 mg of urea was weighed and added to a beaker along with 3 mL of the extraction solution. Then, 10 mL of deionized water was added to the solution and stirred until the urea dissolved. After this step, the solution was transferred into a Teflon liner. The solution was then stirred, and a piece of Ni foam with dimensions of 2 × 3 cm^2^ was placed inside. After 6 h in the furnace at 150 °C, the autoclave was taken out and allowed to cool to ambient temperature. Then, the Ni foam was taken out and washed with distilled water after cooling. Finally, it was dried in an oven at 60 °C for 1 d. The production steps are schematically shown in Figure 1.

CO(NH_2_)_2_ + H_2_O → 2NH_3_ + CO_2_ (2)

NH_3_ + H_2_O → NH_3_·H_2_O → NH_4_^+^ + OH^−^ (3)

Zn^2+^ + 2OH → Zn(OH)_2_ (4)

Zn(OH)_2_ → ZnO + H_2_O (5)

### 2.4. Materials Characterization

The concentrations of Zn after extraction were determined by atomic emission spectrometers (AAS, Thermo Scientific ICE 3400, Waltham, MA, USA) with the flame method. X-ray diffraction (XRD, Rigaku Ultima IV X-ray diffraction, Tokyo, Japan) was utilized to analyse the crystallography of constituents in the electrode after hydrothermal synthesis. Cu-based Ka radiation at a wavelength of 0.1546 nm and diffraction angles from 10° to 90° were used in the XRD measurements. Fourier transform infrared spectroscopy (FTIR, Bruker Alpha, Ettlingen, Germany) was also utilized to analyse the chemical bond structure, while the electronic bond structure and valence states were examined using X-ray photoelectron spectroscopy (XPS, Flex Mod System) with a monochromatic Al X-ray source (Al-K line: 1486.6 eV). The microstructure and morphology of the formations on the Ni foam surface were analysed using high-resolution transmission electron microscopy (HR-TEM, Thermo Scientific Talos F200S, Waltham, MA, USA) and scanning electron microscopy (SEM, Carl Zeiss Ultra Plus, Jena, Germany).

### 2.5. Electrochemical Tests

The ZnO-doped Ni foam electrode was electrochemically examined through cyclic voltammetry (CV), galvanostatic charge–discharge (GCD), and electrochemical impedance spectroscopy (EIS) measurements using the Ametek Parstat 4000, USA device. The measurements were performed in a 6 M KOH solution using a three-electrode setup, with a graphite rod as the counter electrode, an Ag/AgCl reference electrode, and the ZnO-doped Ni foam as the working electrode. For the measurements, the electrolyte solution was transferred to a beaker, and the electrodes were immersed in it. The total surface area of the working electrode immersed in the electrolyte was 0.5 × 1.5 cm^2^. CV measurements were performed in the potential range of 0–0.45 V. GCD tests were conducted in the current range from 1 mA to 7 mA, and EIS tests were carried out in the frequency range from 100 kHz to 0.1 Hz with a bias potential of 0 V and an AC signal amplitude of 10 mV. Following the GCD measurements, the specific capacitance (*Cs*) of the working electrode was calculated according to Equation (6) [31,34,35].

(6) $$Cs=\frac{I\times \Delta t}{\Delta V\times S\;}$$

In this equation, I is the applied current, Δt is the discharge time, ΔV is the applied potential, and S is the surface area of the electrode immersed in the solution. Furthermore, Equations (7) and (8) were applied to calculate the energy and power densities of the working electrode, respectively.

(7) $$E=\frac{Cs\times V^{2}}{7.2}$$

(8) $$P=\frac{3600\times E\;}{\Delta t}$$

## 3. Results

### 3.1. Material Characterization

**Figure 2:**
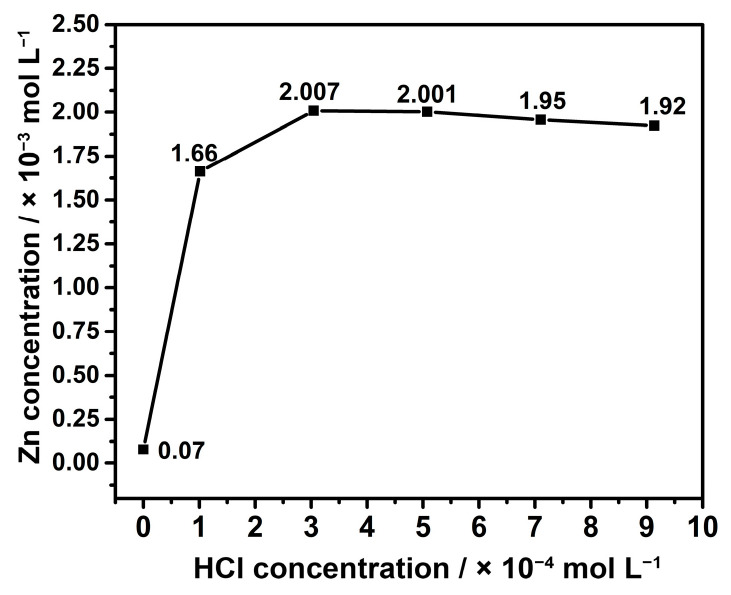
Concentration of Zn in different acid concentrations.

This section focuses on the evaluation of Zn concentrations in the solutions following the extraction process. Initially, the volumes of HCl utilized in the solutions, specifically 1–3–5–7–9 mL, were converted to concentrations of 20–50–90–130–170 ppm, respectively. Afterward, the Zn concentrations in each solution were measured using AAS and presented in Figure 2. The Zn concentration in the leaching solution without HCl, corresponding to pure water, was found to be 5.1 ppm. When the acid concentration was increased to 10 ppm, the Zn concentration in the solution exhibited a significant increase, reaching 108 ppm. Furthermore, upon further increasing the acid concentration to 50 and 90 ppm, the Zn concentrations reached 130.5 and 130.1 ppm, respectively. It was deduced that the solubility of Zn reached its maximum saturation level at this stage. However, raising the acid concentration to 130 and 170 ppm resulted in Zn concentrations remaining around 127 and 125 ppm, respectively. The subsequent decrease in Zn concentration can be attributed to the reformation of compounds involving the boron acid component in the solution, leading to precipitation. These findings highlight that an optimal HCl concentration of approximately 50–90 ppm is suitable for the extraction of Zn metal from zinc borate.

**Figure 3:**
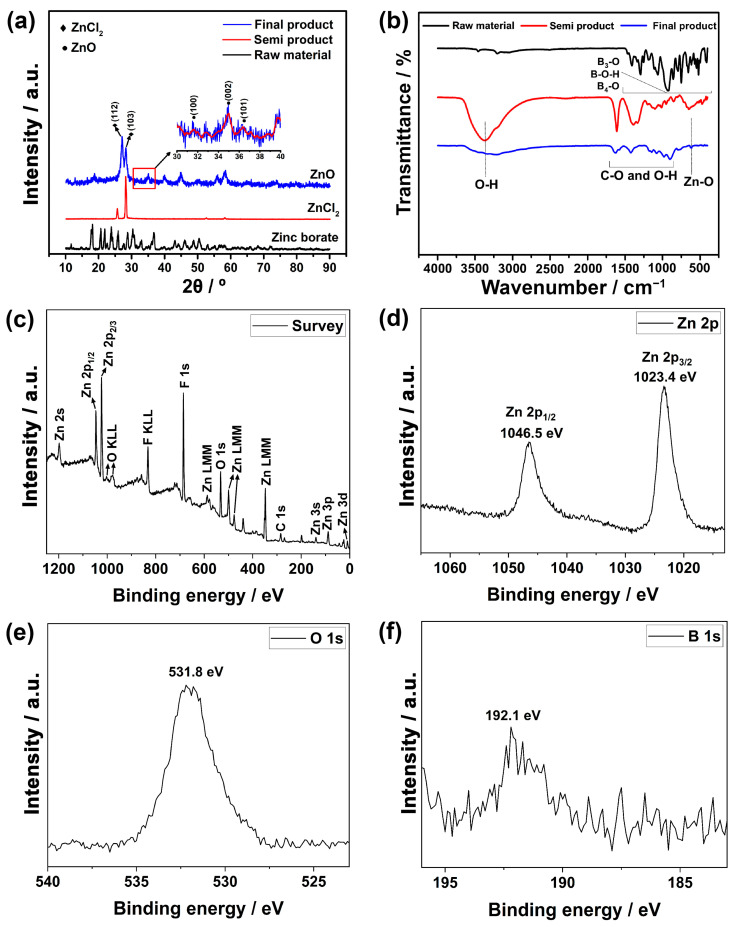
The (**a**) XRD and (**b**) FTIR analysis results of the raw material, semi-finished product, and final product and XPS spectrum of final product: (**c**) survey, (**d**) Zn 2p, (**e**) O 1s. and (**f**) B 1s.

The characterization studies commenced with the analysis of XRD patterns, which are given in Figure 3a. The analysis involved examining the raw material, zinc borate, followed by the semi-finished product obtained after extraction and, finally, the post-synthesis products generated through the hydrothermal technique. The XRD pattern of zinc borate exhibited characteristic peaks within the range of approximately 15° to 70° (JCPDS File No. 35-0433) [36]. In the semi-finished product, two distinct peaks were observed at around 25° and 28°, which corresponded well with the characteristic zinc chloride planes of (112) and (103), respectively [37]. Additionally, peaks corresponding to the (100), (002), and (101) planes were observed at approximately 31°, 34°, and 36°, respectively, indicating the presence of ZnO [38]. Notably, the final product displayed additional peaks, suggesting the retention of some ZnCl_2_ compound within its structure.

In Figure 3b, the FTIR analysis results for interatomic bond characterization are presented. Similar to the XRD analysis, the characteristic B_3_-O, B-O-H, and B_4_O bonds of the raw material zinc borate were observed. In the semi-finished product, observations of O-H bonds and C-O bonds were noted, likely stemming from possible impurities and moisture in the sample. In the final product, the occurrence of Zn-O bonds indicated the presence of ZnO. However, the relatively weak strength of the Zn-O bond is likely attributed to the presence of other impurities [36].

XPS was also conducted to identify the components of the electrode. Figure 3c presents the survey scan results of this analysis. According to this result, the sample was mainly comprised of Zn, O, F, B, and C elements. Zn and O indicate the formation of ZnO in the structure. The presence of low-intensity B at 192.1 eV may originate from the zinc borate used as a raw material during processing. However, the presence of F and C is likely due to additives and residues used in production. Furthermore, in Figure 3d, distinct peaks at approximately 1046 eV and 1023 eV were obtained, corresponding to 2p1/2 and 2p3/2 orbitals of Zn, respectively. Similarly, Figure 3e shows a prominent single peak at approximately 531 eV corresponding to 1s orbital of O. It was determined that the binding energy difference between Zn2p3/2 and Zn2p1/2 emissions was calculated as 23.1 eV, which is a characteristic value for ZnO [39,40].

**Figure 4:**
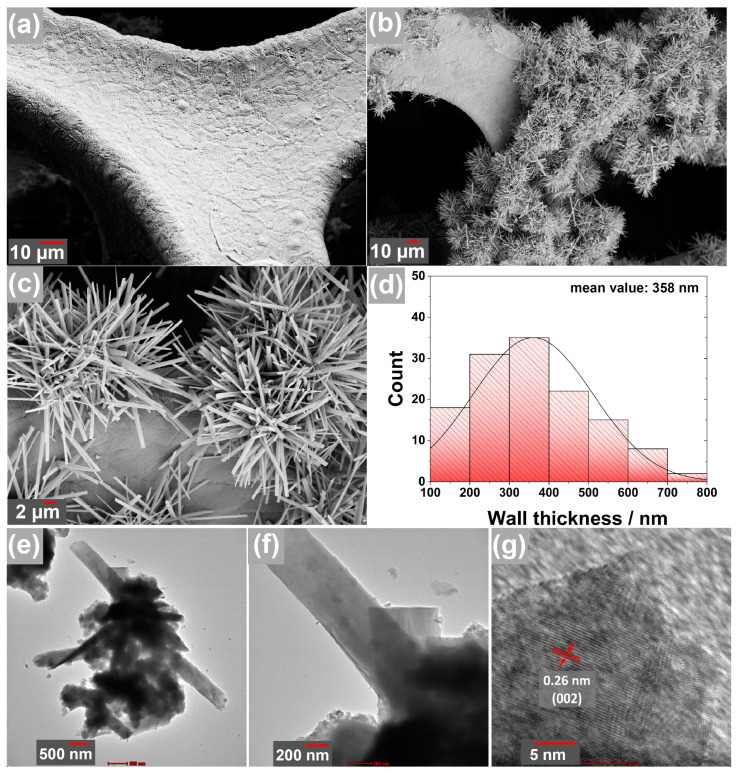
(**a**–**c**) SEM images of synthesized ZnO nanorods in different growth (**d**) distribution histogram plot of nanorods, (**e**–**g**) TEM images of the electrode at various magnifications.

The surface structure of the obtained products through the hydrothermal process on the Ni foam substrate was examined by SEM, as illustrated in Figure 4a–c. The SEM analysis revealed unique formations on the surface of the pure Ni foam, displaying both rod-shaped and strip-shaped structures. These formations can be clearly observed in the overview images presented in Figure 4a,b. Upon closer inspection in Figure 4c, it is evident that these formations possess a strip-like geometry rather than a bar-like structure. To determine their dimensions, the strips were analysed using the ImageJ software, and the results were plotted in Figure 4d. The analysis indicated an average strip size of approximately 358 nm, with less than 10% of the strips measuring around 100 nm. It is noteworthy that the synthesis of ZnO crystals in the form of strips, which deviates from the commonly observed rod-like form reported in existing literature, represents a significant and novel discovery of this study [41,42]. Previous studies have demonstrated that nanostructures formed on current collector surfaces, as observed in this research, can increase the surface area and enhance the ion-holding capacity and electrochemical performance [43,44]. The ZnO formations in this study are, as desired in the literature, quite thin and nanoscale in size. This achievement highlights the potential for enhanced energy storage capacity in such electrode designs.

Detailed examinations of the surface of Ni foam were also carried out using HR-TEM in Figure 4e–g. In this analysis, it was observed that formations with nanoribbon-shaped crystals, as well as heterogeneous structures of different geometries, were present (Figure 4e,f). The significant curvature observed in Figure 4e indicates that these ribbons have very thin walls. The high-resolution image of these ribbons is presented in Figure 4g. The d-space value of 0.26 nm suggests that it corresponds to the (002) plane of ZnO, which was also detected in the XRD patterns. Based on these observations, it can be inferred that the majority of the crystals formed are primarily composed of ZnO.

### 3.2. Electrochemical Properties

**Figure 5:**
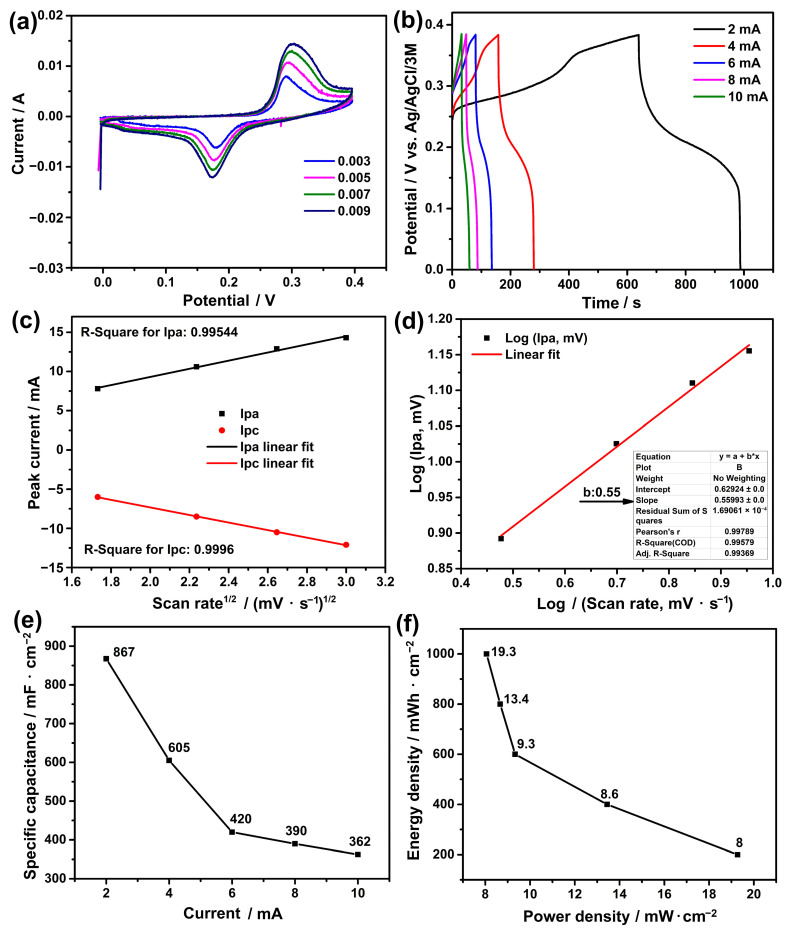
Electrochemical test results: (**a**) cyclic voltammetry, (**b**) galvanostatic charge–discharge, (**c**) Randel–Sevcik, (**d**) electrochemical kinetic, (**e**) specific capacity, and (**f**) energy density vs. power density.

The electrochemical test results are illustrated in Figure 5. The measurements were conducted within the range of 0–0.4 V in accordance with the literature [24,45,46]. CV measurements at various scan rates (Figure 5a) showed that a broad peak was observed at approximately 0.3 V in the anodic region, while a peak around 0.17 V was observed in the cathodic region. These peaks can be attributed to the electron migration and transformation of Zn^2+^ near the p-n heterojunction layer into Zn^3+^. This transformation is associated with the reaction mechanism described in Equation (9) [24].

ZnO + 2OH^−^ ↔ ZnO(OH^−^) + e^−^ (9)

The presence of reversible peaks in both the anodic and cathodic regions suggests favourable reversibility of the reaction, which is a desired characteristic for energy storage systems [47]. The intensity of the peaks is affected by the variables such as scan rate and ion adhesion to the electrode surface. Previous studies have shown that increasing scan rate results in higher peak intensities in a linear relationship [30,31,34]. This trend is confirmed by the slope values of 0.99544 in the anodic region and 0.9996 in the cathodic region, as depicted in the Randel–Sevcik plot shown in Figure 5c. Understanding the charging mechanism of these electrodes is crucial, and it can be inferred from the CV graph. To conclude the type of charging mechanism, the logarithm of the anodic peaks (log(I_p_)) against the logarithm of the scan rates (log(V)) was plotted according to the equation Ipa = a × v^b^, where a and b are variable constants. The slope of this graph, as shown in Figure 5d, helps to determine the value of ‘b’. When the slope is close to 0.5, it indicates a primarily diffusion-controlled mechanism (battery-type behaviour), whereas a value close to 1 suggests capacitive control [48]. Hence, with a ‘b’ value of 0.55, the charging mechanism of the electrode was primarily diffusion-controlled.

Chronoamperometry measurements were carried out for galvanostatic charge–discharge (GCD) analyses. This allows for the calculation of specific capacity, energy, and power densities of the electrode at different current values. The results of the GCD tests are given in Figure 5b. The discharge times of the electrode for currents of 2 mA, 4 mA, 6 mA, 8 mA, and 10 mA were determined to be 347 s, 121 s, 56 s, 39 s, and 29 s, respectively. Accordingly, the specific capacity at these currents was calculated to be 867 mF·cm^−2^, 605 mF·cm^−2^, 420 mF·cm^−2^, 390 mF·cm^−2^, and 362 mF·cm^−2^, as shown in Figure 5e. According to these results, a non-linear decrease was observed in the specific capacitance and energy-power density values with respect to current density. This decrease is directly correlated with the current density that plays a role in the attraction of ions to the electrode. At low currents, ions have sufficient time to adhere to both the surface and interior of the electrode. However, at high currents, ions rapidly move to the electrode surface and form an ion film, preventing more ions from penetrating the interior [49]. This leads to a disproportionate decrease in ion attachment at high currents. The non-linearity of this decrease is likely due to this phenomenon. Similar situations were observed in our previous studies as well [30,34,35]. At the same time, these results were obtained from the ZnO-modified Ni foam electrode. The performance of pure Ni foam without ZnO modification was not included in the comparison, as it would not be notably significant in accordance with the literature [50,51]. Furthermore, the energy (E) densities of the electrode at the same currents were determined as 19.3 mWh·cm^−2^, 13.4 mWh·cm^−2^, 9.3 mWh·cm^−2^, 8.67 mWh·cm^−2^, and 8.05 mWh·cm^−2^, while the power (P) densities were calculated as 200 mWh·cm^−2^, 400 mWh·cm^−2^, 600 mWh·cm^−2^, 800 mWh·cm^−2^, and 1000 mWh·cm^−2^, as depicted in Figure 5f. These findings indicated that the specific capacity and energy density showed a decrease as the current density increased. This behaviour is likely linked to the interaction of ions with the electrode [35,47]. At lower current values, ions can penetrate through the inner parts of the electrode, allowing for efficient energy storage. However, at higher currents, ions tend to collect on the surface, forming an ion layer that restricts their access to the electrode interior. As a result, ion retention, which is essential for energy storage, showed a reduction.

**Figure 6:**
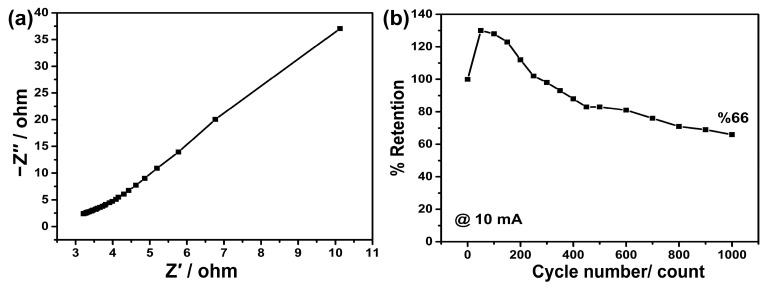
(**a**) EIS and (**b**) multiple charge–discharge (stability) measurements.

Frequency-dependent impedance measurements were conducted to examine the electrochemical interactions between the electrode and the electrolyte, and the results are presented in Figure 6a. In a standard supercapacitor, a semi-circle and a linear region are expected to be observed. However, in this study, the semi-circle region was not pronounced [52]. The starting point of this graph represents the resistance of the solution, which was measured to be approximately 4 ohms. On the other hand, the endpoint of the semi-circle indicates the charge transfer resistance of the electrode. The indistinct semi-circle in this region is likely due to the low charge transfer resistance of the electrode. Low scan rates were preferred in the CV measurements to mitigate the occurrence of high potential jumps as a result of the low charge transfer resistance. The linear region in this graph represents the diffusion resistance of ions to the electrode and is referred to as the Warburg impedance (Zw) [53]. The steep slope of this region indicates low diffusion resistance. The dominance of the diffusion-controlled charge mechanism in the electrode kinetics calculations can be associated with this high slope [54,55].

The stability measurements were performed at a current of 10 mA, and the results are presented as percentage retention in Figure 6b. According to the findings, there was a sharp increase in the retention value, reaching 130% within the first 50 cycles. This increase is likely associated with a higher penetration of ions from the electrolyte to the active regions on the electrode surface. It can be interpreted as an increased wetting of the active material by ions with longer charge–discharge durations. Indeed, this situation has been encountered in numerous previous studies [30,31]. The retention value remained above 100% until the cycle numbers reached 100, 150, 200, and 250. As the cycle numbers increased, the retention value gradually decreased. After 400 cycles, the retention value was around 90%, while it dropped to approximately 80% and 70% after 600 cycles and 900 cycles, respectively, indicating stable behaviour. However, after reaching 1000 cycles, the stability decreased to around 66%. This decline in stability is disadvantageous despite the high specific capacitance of the electrode. However, it could be attributed to the fact that stability measurements were conducted at a high current value of 10 mA. It is possible that lower currents would lead to increased stability, similar to specific capacitance. Therefore, in future studies, it is necessary to repeat the multiple-cycle measurements at various current densities.

Table 1 provides a summary of previous studies conducted to enhance the electrochemical performance of ZnO. In these studies, pure ZnO was generally compared, and its specific capacitance was reported to be approximately in the range of 150–200 F·g^−1^. To improve this performance, carbon-based additives with high surface area and conductivity were introduced. The results showed that the specific capacitance reached a maximum value of 820 F·g^−1^. However, it was observed that even without active materials, the capacitance could only reach around 630 F·g^−1^. The literature suggests that the synthesis of metal oxides with thin-wall structures offers advantages in terms of facilitating ion diffusion. In this context, carbon particles coated with ZnO in a core-shell structure as thin films also exhibited a specific capacitance of 630 F·g^−1^. However, all of these studies could not surpass the specific capacitance values of Fe/Cu/Ni-based metal oxide counterparts [56,57,58]. This was also true for CuFe_2_O_4_ in the literature, where Bandgar et al. synthesized CuFe_2_O_4_ with nanoflake geometry and thin-wall structure, achieving almost the highest performance [59]. Building on these findings, in this study, ZnO was synthesized in a sustainable manner with a thin-strip nanostructure, and its specific capacitance was determined to be 867 mF·cm^−2^. However, a significant disadvantage of this electrode developed through the direct synthesis method is its low cycle life. It is believed that, similar to the other studies, the addition of carbon-like components can improve the cycle life as well.

## 4. Conclusions

The primary aim of this study was to develop high-performance electrodes for supercapacitor applications using ZnO derived from zinc borate minerals. To achieve this, Zn was extracted from zinc borate through a hydrometallurgical method, and the solution with the highest Zn concentration was determined using AAS analysis. Subsequently, ZnO was directly synthesized on the surface of Ni foam current collectors through a hydrothermal approach using the extracted solution. Characterization of the resulting structure analyses revealed the formation of ZnO crystals with a nano-strip pattern and an average thickness of 358 nm. The electrode exhibited a maximum specific capacitance of 867 mF·cm^−2^ at 2 mA, with energy and power densities of 19.3 mWh·cm^−2^ and 200 mW·cm^−2^, respectively. On the other hand, a notable drawback was observed in terms of stability, as the performance decreased to 66% after 1000 cycles. Although the stability of the synthesized electrode may require further enhancement, it showed a promising performance when compared to the existing literature, which can be primarily attributed to the synthesis of nanorods with thin walls. However, the low cyclic stability remains one of the significant issues in this study. This situation could be addressed through experimentation with different manufacturing methods or appropriate measurement conditions for the electrode.

## Figures and Tables

**Table 1:** Comparing the performance of electrodes based on ZnO in previous studies.

Electrode Materials	Electrolyte	Potential (V)	Scan Rate/ Current Density	Specific Capacity (Cs)	% Retention	Ref.
ZnO nanostrips	6 M KOH	0 to 0.4	2 A·g^−1^	867 mF·cm^−2^	~66% after 1000 cycles at 10 mA	This study
ZnO/C	1 M Na_2_SO_4_	0 to 0.8	1 A·g^−1^	820 F·g^−1^	~92% after 400 cycles at 50 mV·s^−1^	[60]
ZnO/C Core-shell	6 M KOH	0 to 0.4	2 A·g^−1^	630 F·g^−1^	~70% after 5000 cycles	[24]
ZnO/GO	1 M Na_2_SO_4_	0 to 1	5 mV·s^−1^	312 F·g^−1^	~95% after 100 cycles at 10 mV·s^−1^	[26]
ZnO/Graphene	1 M KOH	0 to 0.5	3 A·g^−1^	291 F·g^−1^	~88% after 1000 cycles at 4 A·g^−1^	[25]
ZnO/ZnS	1 M KOH	−0.6 to −1.3	1 mA·cm^−2^	217 mF·cm^−2^	~81% after 2000 cycles at 2 mA·cm^−2^	[20]

## Data Availability

All the data is available in this manuscript.
